# General practitioners’ willingness to participate in research networks in Germany

**DOI:** 10.1080/02813432.2022.2074052

**Published:** 2022-06-30

**Authors:** Larissa Virnau, Annett Braesigk, Tobias Deutsch, Alexander Bauer, Eric Sven Kroeber, Markus Bleckwenn, Thomas Frese, Heidrun Lingner

**Affiliations:** a Institute of Medical Psychology, Hannover Medical School, Hannover, Germany; b Department of General Practice, Medical Faculty, University of Leipzig, Leipzig, Germany; c Institute of General Practice and Family Medicine, Martin-Luther-University Halle-Wittenberg, Halle/Saale, Germany

**Keywords:** General practice, primary health care, community-based participatory research, research network, financial compensation

## Abstract

**Objectives:**

To investigate general practitioners’ (GPs’) willingness to participate in long-term medical research and in research networks (RNs).

**Design and setting:**

Cross-sectional survey among German GPs around Halle-Wittenberg and Leipzig in 2020.

**Subjects:**

Random sample of 905 GPs.

**Main outcome measures and results:**

Response rate 37%, 69% female. Overall, 57% were interested in participating in medical research, 34% in an active role in a RN. Interest in RN participation was positively associated with male sex, younger age, previous experiences in medical research, being involved in teaching undergraduates, and having qualification in a further specialty. Main motivators were improving patient care, giving a more realistic picture of GP care, and carrying out research on topics within their own interest areas and a reliable contact person at the leading institution. Most GPs were not afraid of reduced earnings; however, time investment was the main barrier for participation. GPs were willing to dedicate twice as much time to research when remuneration was offered. High rated topics were polypharmacy, chronic diseases, drug safety and adverse drug reactions.

**Conclusion:**

GPs are interested to participate in practice-based research. The study results providing useful and generalizable insights in barriers and motivators should be considered when building and running GP-RNs.

**KEY POINTS**
- There is a difference between general practitioners’ (GPs’) overall interest in clinical research and their job and socio-demographic related readiness to participate in research networks (RNs).
- GPs are interested in RNs when it is a resource of and leading to enhanced patient-oriented care.
- GPs are willing to dedicate twice as much time to research when remunerated.
- GPs need a reliable counterpart within the leading institution.

## Introduction

Despite general practitioners’ (GPs’) key role in Germany’s primary health care, clinical research in general practice is still scarce. Most of the research informing GPs’ clinical decision-making is carried out at in-patient facilities [1], although their results are rarely suited for a direct translation to out-patient settings [2].

Primary care research has been increasingly promoted over the last decades but varies substantially between countries [3]. While in the USA or the UK developing research capacity by building research networks (RNs) is fairly advanced, other countries, like Germany and France, are progressing more slowly [4,5].

Patient care in German general practice is solely carried out in private, outpatient practices [6] and medical university-departments are depending on a tight collaboration with local GPs when teaching or performing research [7].

The German Federal Ministry of Education and Research (BMBF) is currently promoting collaboration efforts by funding RNs like RaPHaeL (Research Practices Halle-Leipzig) [8], a joined project of the universities of Halle-Wittenberg, Leipzig and Hannover. It aims to establish a continuous, high-quality RN with a mandatory research-specific training for GPs and their staff.

More information on GPs’ participation-readiness in practice-based clinical research and in long-term RNs was needed to enhance the recruitment of network members.

## Material and methods

### Sampling and design

**Figure 1.**
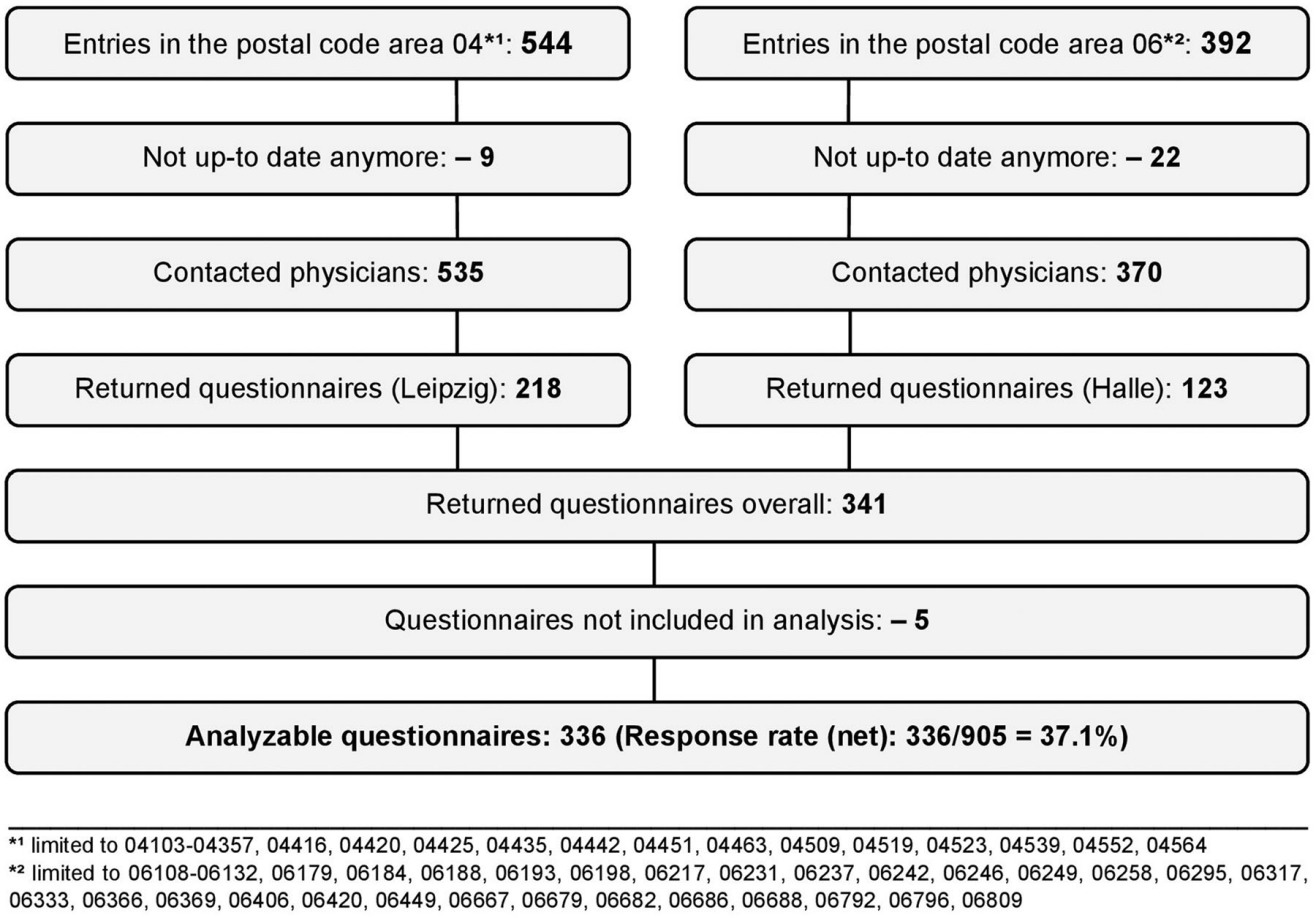
Sampling flowchart.

For this cross-sectional paper-based survey, all GPs practicing in the German postal code areas 04 (Saxony) and 06 (Saxony-Anhalt) were addressed between July and September 2020 (publicly available data-lists as of 16th June 2020; Figure 1). The participants were not provided with any information about the RaPHaeL project or the possibility to adhere to its RN later on. A post-delivered envelope included a formal cover letter, an anonymous questionnaire, and a postage paid return envelope. The completed questionnaires could also be returned by fax. All GPs received a reminder after four weeks including questionnaire and return envelope.

### Patient and public involvement

No patients were involved.

### Non-response analysis

To enable non-response analysis each questionnaire was linked with an anonymized code-list referencing GPs’ sex, degree of qualification, specialization and the mail’s deliverability.

### Questionnaire

Lacking appropriate validated research instruments, a questionnaire was developed by health scientists and GPs based on previous literature-research, pre-tested and adapted corresponding to the Concurrent Think Aloud method ensuring its comprehensibility, suitability, and validity. The answers to the 81 final-questions were single and multiple-choice options and/or free-text fields.

The MORNING-questionnaire (acronym: Medical Research Or Research Network Interest in General Practice) addressed the following topics: socio-demographic and job-related variables, interest in participation in medical research (target variable in logistic regression model 1), active involvement in a RN (target variable in logistic regression model 2), previous research experience, rating of research topics (selection based on literature[1]) and motivating factors and barriers for RN-participation (Supplementary File 1 questionnaire).

### Data analysis

Data was analyzed using IBM SPSS Statistics for Windows, Version 25.0. Single variables frequencies are presented as %(n/n_valid_):

$$\left(n_{valid}\right)All\;=\;\left(n_{valid}\right)interested\;in\;RN\;+\;\left(n_{valid}\right)not\;interested\;in\;RN\;+\;missing\;values\;\left(in\;RN\;interest\right)$$

The same proceeding applies to the item ‘interest in medical research’. Continuous variables are presented as mean ± standard deviation (SD), complemented by the median when appropriate. The following statistical tests were performed: Chi^2^, Kolmogorov Smirnoff, the Mann Whitney U test and binary logistic regression (stepwise forward). Due to the exploratory nature of the study, all variables with *p* < 0.20 (screening criterion) in univariate comparisons were considered for inclusion in the respective regression model. Statistical significance was assumed for *p* < 0.05.

## Results

### Response and non-response

Out of 936 addressed questionnaires, 905 were deliverable and 341 returned. Five were excluded from the analysis since the only answered items were socio-demographic ones (Figure 1). A non-response analysis revealed no statistically significant differences between responders and non-responders regarding sex and specialization (general practice or internal medicine). GPs with an academic degree participated more frequently. Detailed socio-demographic and job-related characteristics are presented in Table 1.

**Table 1.** Socio-demographics and job-related characteristics – total sample and comparison between physicians with and without interest in participating in a research network.

Variable	All	Interested in research network	Not interested in research network
	% (n/n_valid_)^a^	% (n/n_valid_)^a^	% (n/n_valid_)^a^
Female*	69.0 (227/329)	58.7 (61/104)	74.2 (155/209)
Age in years (mean ± SD, median)**	52.2 ± 10.5, 52	47.2 ± 8.9	54.2 ± 10.3
Specialist for general practice (vs. others)	72.4 (231/319)	77.9 (81/104)	70.0 (140/200)
Additional specialty title (vs. none)	13.3 (43/323)	18.3 (19/104)	10.8 (22/204)
Training undergraduates (vs. not)*	30.7 (99/323)	40.0 (42/105)	26.7 (54/202)
Years being a GP (mean ± SD, median)**	16.2 ± 12.2, 12	11.5 ± 10.5	18.1 ± 12.1
Catchment area of the practice: city (vs. town/ rural area)	61.7 (190/308)	63.3 (62/98)	62.4 (121/194)
Medical documentation: electronically (vs. paper-based)**	88.1 (290/329)	95.3 (102/107)	84.0 (173/206)
Experiences with research (vs. none)**	57.1 (192/336)	69.4 (75/108)	51.2 (108/211)
Work satisfaction*			
Very satisfied	33.8 (112/331)	46.2 (49/106)	27.9 (58/208)
Rather satisfied	56.2 (186/331)	44.3 (47/106)	61.1 (127/208)
Rather or very dissatisfied	10.0 (33/331)	9.4 (10/106)	11.1 (23/208)
Economic satisfaction			
Very satisfied	32.8 (109/332)	36.2 (38/105)	31.4 (66/210)
Rather satisfied	53.9 (179/332)	48.6 (51/105)	55.7 (117/210)
Rather or very dissatisfied	13.3 (44/332)	15.2 (16/105)	12.9 (27/210)

*Note*: (n_valid_)All = (n_valid_)interested in research network + (n_valid_)not interested in research network + missing values. ^a^Unless otherwise indicated. Group differences between interested and not interested in research network are indicated as follows: *p < 0.05, **p < 0.005.

### Willingness to participate in research

More than half of GPs (57%) indicated general interest in participating in medical research, 34% could imagine playing an active role in a RN. The results contrasting the socio-demographic and job-related characteristics of GPs with and without interest in a RN are presented in Table 1. The following characteristics were associated with more frequent interest in a RN: male, younger age, involved in teaching undergraduates, performing medical documentation electronically, high work satisfaction and more experiences in medical research. Detailed results regarding GPs’ general interest in medical research may be found in Supplementary File 2.

### Previous experiences with research

Most participating GPs have been previously engaged in some kind of medical research activities (Table 1): completing questionnaires (96%), providing patient data (79%) and participating in interviews or group discussions (51%). Very few physicians (6%) had already initiated their own research projects.

### Motivating factors, important frame conditions and barriers

GPs’ motivations for RN-participation are displayed in Table 2. The items showing the highest impact were: helping to improve patient care, carrying out research within areas of interest, and giving a more realistic picture of GP care. Physicians interested to participate in a RN rated all investigated factors as more motivating (significantly higher), than not interested GPs did.

**Table 2.** GPs' perceptions on what would motivate them to participate in research – total sample and comparison between physicians with and without interest in a research network.

Variable	All	Interested in research network	Not interested in research network
	(Mean ± SD)	(Mean ± SD)	(Mean ± SD)
Improving their patient care**	3.0 ± 1.1	3.5 ± 0.8	2.7 ± 1.2
Carrying out research on topics within their areas of interest**	2.9 ± 1.1	3.4 ± 0.8	2.6 ± 1.2
Giving a more realistic picture of GP care**	2.9 ± 1.2	3.3 ± 0.9	2.7 ± 1.2
Added value for their patients**	2.6 ± 1.2	3.2 ± 0.9	2.3 ± 1.2
Easily plannable scope of work for the research practice network**	2.7 ± 1.2	3.2 ± 0.8	2.4 ± 1.2
Separate remuneration for required working time**	2.6 ± 1.3	3.0 ± 1.2	2.4 ± 1.3
Reimbursement for additional costs, e.g. for training of staff, travel costs etc.**	2.6 ± 1.3	2.9 ± 1.1	2.4 ± 1.4
Exchange between and feedback from colleagues, e.g. on rare diseases**	2.5 ± 1.3	2.9 ± 1.1	2.3 ± 1.3
Processing of practice data allowing the use for own purposes**	2.3 ± 1.2	2.8 ± 1.0	2.1 ± 1.3
Acquiring additional training credit points through participation*	2.4 ± 1.3	2.6 ± 1.3	2.2 ± 1.3
Free or facilitated access to relevant specialist literature**	2.1 ± 1.3	2.6 ± 1.1	1.9 ± 1.4
Patients’ wish for the practice’s participation**	1.9 ± 1.3	2.5 ± 1.1	1.6 ± 1.2
Research workshops within the network**	2.0 ± 1.3	2.4 ± 1.2	1.8 ± 1.2
Official certification as a research practice affiliated to the university**	1.4 ± 1.3	2.2 ± 1.3	1.0 ± 1.2
Possibility of obtaining another academic title**	1.2 ± 1.5	1.9 ± 1.7	0.9 ± 1.3
Mentioning their names in publications**	1.0 ± 1.3	1.5 ± 1.4	0.7 ± 1.0

*Note*: range from 0 = ‘no increase in motivation’ to +4 = ‘very high increase in motivation’; group differences between interested and not interested in research network are indicated as follows: *p < 0.05, **p < 0.005.

Table 3 shows participants’ ratings on the importance of practice-related conditions increasing attractiveness of research and displays the respective differences depending on the stated RN-interest. GPs most frequently rated the following themes as ‘very’ or ‘rather’ important enhancers for the attractiveness of medical research: a direct and reliable contact person at the university, low effort (timewise) for medical doctors and the practice staff, as well as compact updates on practice relevant topics. Training opportunities in research are currently perceived as less attractive motivators for RN-participation.

**Table 3.** GPs' perceptions on what would enhance the attractiveness of medical research in general practice – total sample and comparison between physicians with and without interest in participating in a research network.

Variable	All	Interested in research network	Not interested in research network
	% (n/n_valid_)	%(n/n_valid_)	%(n/n_valid_)
Direct and reliable contact person at the university	90.7 (284/313)	93.4 (99/106)	89.1 (171/192)
Low effort (timewise) for the practice team	92.5 (295/319)	89.7 (96/107)	93.8 (183/195)
Low effort (timewise) for me as a medical doctor*	92.5 (296/320)	87.9 (94/107)	94.9 (186/196)
Compact updates on practice-relevant topics	87.4 (271/310)	87.7 (93/106)	86.2 (163/189)
Timely and practical processing of study results for participating practices*	77.8 (242/311)	84.0 (89/106)	73.4 (141/192)
Seasonal adjustment to the practice workload	75.6 (236/312)	77.6 (83/107)	73.2 (139/190)
Training opportunities in research	59.0 (186/315)	65.4 (70/107)	53.9 (104/193)
Access to the shared, anonymized project database*	51.9 (161/310)	62.9 (66/105)	46.9 (90/192)

*Note*: percentages of participants who consider the presented factors ‘rather important’ or ‘very important’, versus ‘rather unimportant’ and ‘not at all important’; (n_valid_)All = (n_valid_)interested in research network + (n_valid_)not interested in research network + missing values; group differences between interested and not interested in research network are indicated as follows: *p < 0.05).

GPs assessments of potential barriers for RN-participation are shown in Table 4. GPs seemed worried network research activities might increase their daily working time, also adding time pressure on the practice team. ‘Financial losses’ were the least relevant barrier for active network participation. GPs interested in RN-participation perceived nearly all barriers as significantly less relevant than GPs not-interested in participation.

**Table 4.** Potential barriers regarding an involvement in medical research – total sample and comparison between physicians with and without interest in participating in a research network.

Variable	All	Interested in research network	Not interested in research network
	%(n/n_valid_)	%(n/n_valid_)	%(n/n_valid_)
Increase of their daily working time**	90.6 (298/329)	81.3 (87/107)	95.1 (195/205)
Stressing the doctor’s time too much**	82.3 (270/328)	63.6 (68/107)	91.2 (186/204)
Putting too much time pressure on the practice team**	82.3 (270/328)	63.6 (68/107)	91.7 (187/204)
Diminishing the number of patient treatments**	59.1 (192/325)	43.0 (46/107)	67.3 (136/202)
Disruption of their working routine**	58.9 (192/326)	34.6 (37/107)	69.8 (142/202)
Fear of insufficient scientific skills	34.4 (111/323)	27.1 (29/107)	37.1 (75/202)
Fear of insufficient current knowledge**	32.5 (105/323)	20.6 (22/107)	38.3 (77/201)
Financial losses by participation*	13.7 (44/321)	7.5 (8/106)	17.0 (34/200)

*Note*: percentages of participants who ‘rather agree’ or ‘completely agree’ with the presented statements, versus ‘rather disagree’ and ‘completely disagree’; (n_valid_)All = (n_valid_)interested in research network + (n_valid_)not interested in research network + missing values; group differences between interested and not interested in research network are indicated as follows: *p < 0.05, **p < 0.005).

GPs were willing to dedicate 1.5 ± 1.5 h/week (H/W) to research without any remuneration and more than double with financial compensation of 50 ± 28.2 Euros/H (net). Furthermore, GPs willing to allocate non-medical staff for unpaid research purposes for 1.2 ± 1.7 H/W and remunerated 3.4 ± 4.2 H/W.

### Currently important research topics

The GPs’ ratings of research topics to be addressed in general practice are displayed in Supplementary File 3. Polypharmacy, chronic diseases, drug safety and adverse drug reactions were rated as the currently most important ones, while practice management, digitalization/telemedicine and rare diseases were regarded as the least important topics.

### Variables independently associated with research interest

Previous experiences in medical research, younger age, and male sex were independently positively associated with a general interest in participating in medical research (Model 1) and a RN (Model 2) as were current collaboration with the university in training undergraduates and having an additional specialty title. More detailed results are displayed in Table 5.

**Table 5.** Logistic regression analyses predicting the interest in participating in medical research (Model 1) and in a research network (Model 2).

	Model 1 (*N* = 278)		Model 2 (*N* = 273)	
	(Nagelkerkes *R*² = 0.251)		(Nagelkerkes *R*² = 0.238)	
Variables included in the model (stepwise forward LR)	Odds ratio OR (95% CI)	*p*	Odds ratio OR (95% CI)	*p*
Previous experiences with medical research (vs. none)	3.49 (2.03–6.00)	<0.001	2.20 (1.21–4.00)	0.010
Age (in years)	0.94 (0.91–0.96)	<0.001	0.92 (0.90–0.95)	<0.001
Male (vs. female)	2.61 (1.42–4.78)	0.002	1.89 (1.05–3.39)	0.033
Training undergraduates (univ. associated practice) (vs. not)			1.98 (1.06–3.68)	0.032
Additional specialty title (vs. none)			2.19 (1.02–4.68)	0.043

Further variables considered (but not included in the model): documentation in the practice (entirely and mostly electronically vs. entirely and mostly paper-based), work satisfaction (very and rather satisfied vs. rather and very dissatisfied), specialty field (general practice vs. others). Variables not considered despite univariable differences: years being a GP due to high correlation with age (*r* = .84).

## Discussion

### Statement of principal findings

More than half of all GPs were predisposed to participating in medical research. About one-third of them were willing to be actively involved in a RN. Interest in RN-participation was positively associated with the following: male sex, younger age, previous experiences in medical research, being involved in teaching undergraduates, and having qualification in a further specialty. Improving patient care, giving a more realistic picture of GP care, and performing research on topics within their own interest areas were identified as the main motivating factors for network attendance. A reliable contact person at the university in charge enhances the attractiveness of research for GPs. Most GPs were not afraid of reduced earnings; however, time was seen as the main barrier for participation.

### Willingness to participate in medical research or in a RN

To our knowledge, this study was the first of its kind to differentiate between GPs’ general research interest and their willingness to actively participate in a long-term RN. We assessed promising interest in conducting medical research, and less in participating in a predefined RN. This may refer to the anticipation of an increased workload, time investment, or the GPs’ inexperience in RNs.

The importance of work satisfaction among GPs and their team has been observed before [9]. Our findings confirm this relationship assigning this factor a strong RN-predicting power: GPs with high work satisfaction are more likely open to research activities.

Our findings suggest interest in research (network) participation to be positively associated with male gender, (inconsistently discussed in literature [10–12]) and younger age [11–13]. In Germany, more than half of GPs are female following a sharp increase of medical students and female physicians within the last decade [14], reflected in the percentage of female study participants. Given similar developments internationally [15], it is of particular importance to evaluate gender-specific needs and preferences in research. One approach to enhance women’s participation could be to promote working as part of a general practice team, a model promoted by women involved in research [10]. Referring to younger age, GPs nearing retirement might be more hesitant to invest time in research-purposes. Medical research might become more accepted among all age groups when increasing RN-development [8].

In our survey, the willingness to participate in a RN was also positively associated with experiences in training undergraduates. GPs’ interaction with medical students was described as an important introduction to research [16] and may thus have the potential to boost GPs’ further research-involvement. German general practice departments are currently carrying out most of their research in cooperation with local GPs, who are involved in teaching undergraduates [7]. It was stated that efficient research requires a comprehensive establishment of a RN and a research specific training for participating GPs [17].

### Research topics of interest

In line with several previous publications, our data suggest that carrying out research on topics of personal interest is a highly motivating for GPs [11–13,17]. In a newly established RN, the relevance of the research topic for daily clinical practice turned out to be one of the main reasons for GPs involvement [18].

A continuous interaction between general practice departments and GPs is important to identify topics relevant to GPs and their patients [4], in order to define research themes in a top-down approach while simultaneously facilitating a bottom-up selection process over an extended period of time.

In our study, polypharmacy, chronic diseases, drug safety and adverse drug reactions were rated as the most relevant research topics. This might be due to their importance in the multimorbidity context especially when dealing with rising prevalence of chronic conditions in both genders, a given in various European countries [19]. Multimorbidity being a risk factor for excessive polypharmacy [20], older adults (>70 years) are most concerned and at risk for adverse drug reactions. The tight involvement of German GPs with these patients adding to the lack of corresponding practical guidelines might explain the pronounced interest of GPs in the polypharmacy-topic in our cohort.

Clinical studies on common complaints in non-selected patients have already been defined as a priority in European general practice research agenda in 2010 [1], further stressing the high importance of research on common, everyday issues in the real life setting of primary care and connecting RNs and the overarching international primary care research agenda [21].

### Motivating factors and barriers in network-based clinical research

Helping to improve patient care was stated as the main motivator for RN-participation [18,22]. Interaction between GPs in RNs could facilitate reflections on their own practice behavior and promote clinical changes, followed by improved care [22].

Although an official certification as a ‘research practice affiliated to the university’, and the possibility of obtaining an academic title were less motivating for network-participation, there was a significantly higher interest in university affiliation in network-aspirants than in GPs declining network-cooperation. Possibly, network-interested GPs have a more positive perception of universities due to a personal connection or teaching activities [16]. The reputation of the institution and experiences in previous cooperation are important aspects in GPs’ recruitment [23]. Second, a certificate ‘university-affiliated research practice’ allows to enhance their ‘public image’ [24].

Good communication throughout all project phases and with all the team involved is particularly important to facilitate or improve practice-based research [22]. Moreover, a reliable contact person at the university seems to be a condition *sine qua non* fora strong relationship between GPs and the university. Expertise along with research training was perceived necessary all over Europe in order to improve research capacity at an individual level [25].

Rather few participants were uncertain about their scientific skills and their current knowledge. This may partly explain the low rating for ‘training opportunities in research’. The majority of GPs endorsed compact updates on research-relevant topics allowing for easily accessible expert information, helping practices stay up-to-date, and fostering learning and exchange within the network.

The amount of time spent on an activity during consultation hours is highly relevant for GPs and a major barrier to research interest [12,26]. However, network-candidates consider time spent in network activities a minor impediment which might be due to a more positive attitude towards or a higher affinity to research. Generally, a low time-effort for GPs and their staff will positively affect the attractiveness of research, and might thus improve future recruitment [27].

Even though the majority of GPs are not concerned about financial losses due to their time dedicated to research participation, GPs and their staff would appreciate an allowance. With an average of 50 Euros/h, the remuneration perceived as adequate is rather moderate when comparing to the payment offered by the pharmaceutical industry. This is an encouraging aspect as it makes funding rather achievable for academic departments and network-initiators. Currently, GPs of our RN (government-funded by the BMBF) will receive an allowance per enclosed patient when participating in the first RN-survey. With pay, the time GPs would be willing to invest increased accordingly by more than two demonstrating how time is an ‘elastic resource’ for GPs prone to financial compensation [28]. GPs would also appreciate a reimbursement for additional costs enabling continuity and sustainability in research networks [29]. In Norway, RN-participants receive an annual payment to compensate for ongoing administrative work, on top of the hourly remuneration for each study participation [30]. Similarly, in the RaPHaeL-network a financial incentive of 1000 €will be offered for completion of the research curriculum.

### Strengths and weaknesses of the study

This study provides important insights into research-associated motivators and barriers, in addition to GPs characteristics and expectations regarding RN-participation. The findings will be helpful in RN-development geared to the target groups’ needs, while also optimizing the recruitment process of network-participants. The considerable sample size and the study-involvement of GPs from different areas and settings increase the explanatory power and support generalizability of our findings.

Nevertheless, the following limitations should be considered. Firstly, a non-responder analysis indicated a selection bias regarding scientific qualification. This might, together with socially desirable behavior, lead to an overestimation of GPs’ research-interest, and thus limit some findings’ generalizability. Secondly, we only addressed GPs and not the practice staff, despite their central role in patient recruitment for medical studies. Their needs should be subject to further research. Thirdly, some results may be specific to the German areas Saxony and Saxony-Anhalt. Fourthly, the cross-sectional design of this study does not allow causal statements, which should be kept in mind when interpreting the associations found in our logistic regression analyses. We did not adjust for multiple testing taking the exploratory and hypothesis-generating character of the study into account. Finally, we only assessed the voiced intention of research interest. To what extent this interest ultimately translates into real research participation is yet to be assessed.

## Conclusion

A substantial number of GPs are interested in participating in research and practice-based RNs. The lower interest in RNs of female GPs should be subject of further research, especially since the proportion of female physicians is increasing in many countries [15,31]. A specific promotion and encouragement of women regarding scientific activities during undergraduate and postgraduate medical education could be beneficial.

Moreover, future efforts should promote and prioritize practice-driven research topics and enable GPs to perform them while ascertaining a low and predictable time effort for GPs and practice staff, and a reliable contact person at university. Although GPs are not afraid of financial losses, adequate (rather moderate) remuneration clearly increases the time they are willing to invest.

Our findings are relevant for the development of primary care research and RNs in general practice settings on a national level and may guide recruitment strategies and constituting networks in other countries as well.

## Supplementary Material

Supplemental Material

Supplemental Material

Supplemental Material

## Data Availability

The data may be obtained from the authors upon reasonable request.
