# Supplementary material for: General practitioners’ willingness to participate in research networks in Germany
Source: Scand J Prim Health Care. 2022 Jun 30;40(2):237–45. doi: 10.1080/02813432.2022.2074052 (PMC9397419; doi:10.1080/02813432.2022.2074052)
Supplement: Supplemental Material [file IPRI_A_2074052_SM1880.docx]

**
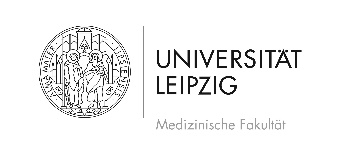
**

**
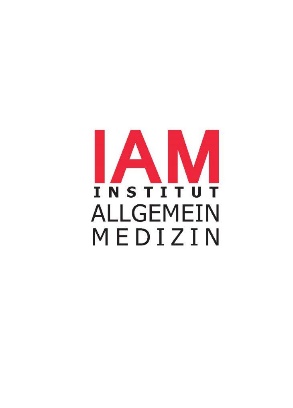

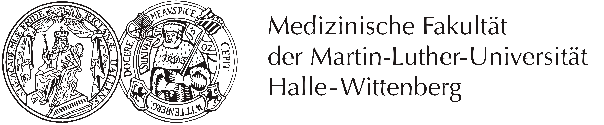
Medical Research Or Research Network Interest in General Practice (MORNING)**

| How would you describe your workplace? | | | | | |
| --- | --- | --- | --- | --- | --- |
| Type of employment contract: | □ employed | | □ self-employed | | |
| Legal structure of the practice: | □ single practice | □ practice sharing | □ joint practice | | □ medical care centre |
| How would you describe the catchment area of your practice? | | □ city | □ town | | □ rural area |
| How is the medical documentation recorded in your practice? | | □ entirely electronically | | □ mostly electronically | |
|  |  | □ entirely paper-based | | □ mostly paper-based | |
| How many hours are you working per week on average (effective working time)? | | | __ __ hours/ week | | |

| What are your experiences with research in your practice? | | | |
| --- | --- | --- | --- |
| *Multiple responses allowed.* | **yes, personally** | **yes, the entire practice team** | **no** |
| Do you have any experience in participating in medical research projects? | □ | □ | □ |
| **If yes:** | | | |
| I have already completed questionnaires. | □ | □ | □ |
| I have already participated in interviews and group discussions. | □ | □ | □ |
| I have already provided patient data. | □ | □ | □ |
| I have already initiated my own research project. | □ | □ | □ |
| Others: | | | |

| Which of the following research topics would you currently consider as particularly important for general practitioners? | | | | |
| --- | --- | --- | --- | --- |
|  | **not at all important** | **rather unimportant** | **rather important** | **very important** |
| Acute diseases | □ | □ | □ | □ |
| Chronic diseases | □ | □ | □ | □ |
| Rare diseases | □ | □ | □ | □ |
| Emergency situations | □ | □ | □ | □ |
| Palliative care | □ | □ | □ | □ |
| Drug safety and adverse drug reactions | □ | □ | □ | □ |
| Polypharmacy | □ | □ | □ | □ |
| Addictive disorders and drug abuse | □ | □ | □ | □ |
| Preventive measures | □ | □ | □ | □ |
| Patient training | □ | □ | □ | □ |
| External influences on health (social, cultural, environmental) | □ | □ | □ | □ |
| Doctor-patient relationship *(e.g., communication, compliance)* | □ | □ | □ | □ |
| Medical decision-making *(regarding examination, diagnosis, therapy)* and prioritization | □ | □ | □ | □ |
| Digitalization, telemedicine | □ | □ | □ | □ |
| Practice management | □ | □ | □ | □ |
| Delegation of medical tasks to non-medical staff | □ | □ | □ | □ |
| Further suggestions for current priority research topics: | | | | |

| **Would you like to participate in medical research?** | □ yes | □ no |
| --- | --- | --- |
| **Can you imagine playing an active role in a network of research practices?** *(Network of practices that are specifically trained for research in a general practitioner context and deal with primary care research topics)* | □ yes | □ no |

| How much time could you imagine participating in research? | |
| --- | --- |
| How many hours would you personally spend on medical research? | a) without remuneration: _______ hours per week |
|  | b) with remuneration: _______ hours per week |
| How many hours would you release your non-medical staff from other tasks for medical research? | a) without remuneration: _______ hours per week |
|  | b) with remuneration: _______ hours per week |
| What, in your view, would be an appropriate remuneration? | ______ Euros per hour (net) for medical staff  ______ Euros per hour (net) for non-medical staff |

| What would motivate you to participate in a research practice network? It would motivate me if…  (possible answers from 0=”no increase in motivation” to +4=”very high increase in motivation”) | | | | | |
| --- | --- | --- | --- | --- | --- |
|  | **0** | **+1** | **+2** | **+3** | **+4** |
| … it would help improve my patient care. | □ | □ | □ | □ | □ |
| … it would encourage exchange between and feedback from colleagues, e.g., on rare diseases. | □ | □ | □ | □ | □ |
| … the scope of work for the research practice network could be easily planned. | □ | □ | □ | □ | □ |
| … it would give a more realistic picture of GP care. | □ | □ | □ | □ | □ |
| … additional costs, e.g., for training of staff, travel costs etc., would be reimbursed. | □ | □ | □ | □ | □ |
| … research workshops would be offered in this context. | □ | □ | □ | □ | □ |
| …I could acquire additional training credit points through participation. | □ | □ | □ | □ | □ |
| … the access to relevant specialist literature would be free or facilitated. | □ | □ | □ | □ | □ |
| … research would be carried out on topics within my areas of interest. | □ | □ | □ | □ | □ |
| … there would be the possibility of obtaining another academic title. | □ | □ | □ | □ | □ |
| … my name would be mentioned in publications. | □ | □ | □ | □ | □ |
| … the practice could be officially certified as a research practice affiliated to the university. | □ | □ | □ | □ | □ |
| … my patients would also wish for us to participate. | □ | □ | □ | □ | □ |
| … there would be an added value for my patients. | □ | □ | □ | □ | □ |
| … the required working time would be remunerated separately. | □ | □ | □ | □ | □ |
| … the processing of practice data would allow the use for own purposes. | □ | □ | □ | □ | □ |

| What would enhance the attractiveness of medical research in general medicine for you? | | | | |
| --- | --- | --- | --- | --- |
|  | **not at all important** | **rather unimportant** | **rather important** | **very important** |
| Low effort (timewise) for me as a medical doctor | □ | □ | □ | □ |
| Low effort (timewise) for the practice team | □ | □ | □ | □ |
| Seasonal adjustment to the practice workload | □ | □ | □ | □ |
| Direct and reliable contact person at the university | □ | □ | □ | □ |
| Training opportunities in research | □ | □ | □ | □ |
| Compact updates on practice-relevant topics | □ | □ | □ | □ |
| Timely and practical processing of study results for participating practices | □ | □ | □ | □ |
| Access to the shared, anonymized project database | □ | □ | □ | □ |
| Other factors increasing attractiveness: | | | | |

| Potential barriers regarding an involvement in medical research | | | | |
| --- | --- | --- | --- | --- |
| *To what extent do you agree with the following statement?* | **completely disagree** | **rather disagree** | **rather agree** | **completely agree** |
| I am afraid that, as a doctor, participating in medical research would stress my time too much. | □ | □ | □ | □ |
| I am afraid that participating in research would put too much time pressure on the practice team. | □ | □ | □ | □ |
| I am afraid that participating in research will increase my daily working time. | □ | □ | □ | □ |
| I am afraid that participating in research would disrupt my working routine too much. | □ | □ | □ | □ |
| I am afraid that my participation in research might diminish the number of patient treatments. | □ | □ | □ | □ |
| I am uncertain whether my scientific skills are sufficient for participating in research. | □ | □ | □ | □ |
| I am uncertain whether my current knowledge is sufficient for participating in research. | □ | □ | □ | □ |
| I am afraid of financial losses by participating in research. | □ | □ | □ | □ |
| Other barriers: | | | | |

| Training, professional and economic satisfaction | | | | |
| --- | --- | --- | --- | --- |
| How do you generally rate your work satisfaction? | □ very dissatisfied | □ rather dissatisfied | □ rather satisfied | □ very satisfied |
| How do you generally rate your economic satisfaction? | □ very dissatisfied | □ rather dissatisfied | □ rather satisfied | □ very satisfied |
| Do you participate in further training beyond the mandatory program (250 CME points/year)? | □ no | □ rather no | □ rather yes | □ yes |

| How would you describe your professional career? | | | | | | | | | | |
| --- | --- | --- | --- | --- | --- | --- | --- | --- | --- | --- |
| Achieved academic title: | □ habilitation | □ doctorate | | | □ diploma | | | | □ none | |
| Medical specialty titles:  Specialist in ______________________________, since: _________ (year)  Specialist in ______________________________, since: _________ (year) | | | | | | | | | | |
| Memberships: | □ DEGAM | | □ Hausärzteverband | | | □ others: _________ | | | | □ none |
| Are you cooperating with the university in training undergraduates? | | | | □ yes | | | □ no | | | |
| Since when have you been a GP? | | | | ________ (year) | | | | | | |
| Are you currently working in your own practice? | | | | □ yes, since: ________ (year) | | | | □ no | | |

| Personal information | | | |
| --- | --- | --- | --- |
| Age: | | __ __ years | |
| Sex: | □ female | □ male | □ diverse |

**Thank you very much for taking the time to complete our questionnaire!**

**We are looking forward to your comments, wishes, and ideas.**

___________________________________________________________________________________________________________________________________________________________________________________________________________________________________________________________________________________________________________________________

**We will happily send you the results of this survey. To do so, please send an email to xxx.**
