# Supplementary material for: General practitioners’ willingness to participate in research networks in Germany
Source: Scand J Prim Health Care. 2022 Jun 30;40(2):237–45. doi: 10.1080/02813432.2022.2074052 (PMC9397419; doi:10.1080/02813432.2022.2074052)
Supplement: Supplemental Material [file IPRI_A_2074052_SM1879.docx]

**SUPPLEMENTARY FILE 2**

| **Supplement Table 1** Socio-demographics and job-related characteristics – comparison between physicians with and without interest in participating in medical research.  (note: (n_valid_)All = (n_valid_)interested in medical research + (n_valid_)not interested in medical research + missing values;  ^+^unless otherwise indicated;  group differences between interested and not interested in medical research are indicated as follows: *p<0.05, **p<0.005) | | |
| --- | --- | --- |
| Variable | Interested in medical research %(n/n_valid_) **^+^** | Not interested in medical research %(n/n_valid_) **^+^** |
| Female ** | 61.9 (109/176) | 78.0 (103/132) |
| Age in years (mean ± SD, median) ** | 49.2 ± 10.1 | 55.6 ± 10.0 |
| Specialist for general practice (vs. others) | 73.0 (127/174) | 74,4 (93/125) |
| Additional specialty title (vs. none) | 15.4 (27/175) | 10.9 (14/128) |
| Training undergraduates (vs. not) * | 37.1 (65/175) | 23.6 (30/127) |
| Years being a GP (mean ± SD, median) ** | 13.2 ± 10.8 | 20.0 ± 12.7 |
| Catchment area of the practice: city (vs. town/ rural area) | 65.5 (110/168) | 57.5 (69/120) |
| Medical documentation: electronically (vs. paper-based) * | 92.1 (164/178) | 84.0 (110/131) |
| Experiences with research (vs. none) ** | 69.4 (125/180) | 42.2 (57/135) |
| **Work satisfaction *** |  |  |
| very satisfied | 30.1 (71/177) | 24.8 (33/133) |
| rather satisfied | 50.3 (89/177) | 63.2 (84/133) |
| rather or very dissatisfied | 9.6 (17/177) | 12.0 (16/133) |
| **Economic satisfaction** |  |  |
| very satisfied | 37.3 (66/177) | 28.4 (38/134) |
| rather satisfied | 48.0 (85/177) | 59.0 (79/134) |
| rather or very dissatisfied | 14.7 (26/177) | 12.7 (17/134) |

| **Supplement Table 2** GPs' perceptions on what would motivate them to participate in research – comparison between physicians with and without interest in medical research.  (note: range from 0 = 'no increase in motivation' to +4 = 'very high increase in motivation';  group differences between interested and not interested in medical research are indicated as follows: *p<0.05, **p<0.005) | | |
| --- | --- | --- |
| Variable | Interested in medical research (mean ± SD) | Not interested in medical research (mean ± SD) |
| Carrying out research on topics within their areas of interest ** | 3.3 ± 0.9 | 2.3 ± 1.2 |
| Improving their patient care ** | 3.3 ± 0.8 | 2.5 ± 1.3 |
| Giving a more realistic picture of GP care ** | 3.2 ± 0.9 | 2.5 ± 1.4 |
| Added value for their patients ** | 3.0 ± 1.0 | 2.0 ± 1.3 |
| Easily plannable scope of work for the research practice network ** | 3.0 ± 0.9 | 2.1 ± 1.4 |
| Separate remuneration for required working time ** | 2.9 ± 1.2 | 2.1 ± 1.4 |
| Reimbursement for additional costs, e.g., for training of staff, travel costs etc. ** | 2.9 ± 1.1 | 2.1 ± 1.4 |
| Exchange between and feedback from colleagues, e.g., on rare diseases ** | 2.8 ± 1.1 | 2.1 ± 1.3 |
| Processing of practice data allowing the use for own purposes ** | 2.7 ± 1.1 | 1.8 ± 1.3 |
| Acquiring additional training credit points through participation * | 2.6 ± 1.2 | 2.1 ± 1.5 |
| Free or facilitated access to relevant specialist literature ** | 2.5 ± 1.2 | 1.6 ± 1.4 |
| Research workshops within the network ** | 2.3 ± 1.2 | 1.5 ± 1.2 |
| Patients’ wish for the practice’s participation ** | 2.3 ± 1.1 | 1.3 ± 1.3 |
| Official certification as a research practice affiliated to the university ** | 1.9 ± 1.3 | 0.7 ± 1.0 |
| Possibility of obtaining another academic title ** | 1.6 ± 1.6 | 0.6 ± 1.1 |
| Mentioning their names in publications ** | 1.3 ± 1.3 | 0.4 ± 0.9 |

| **Supplement Table 3** GPs' perceptions on what would enhance the attractiveness of medical research in general practice – comparison between physicians with and without interest in participating in medical research.  (note: percentages of participants who consider the presented factors 'rather important' or 'very important', versus 'rather unimportant' and 'not at all important';  (n_valid_)All = (n_valid_)interested in medical research + (n_valid_)not interested in medical research + missing values;  group differences between interested and not interested in medical research are indicated as follows: *p<0.05, **p<0.005) | | |
| --- | --- | --- |
| Variable | Interested in medical research %(n/n_valid_) | Not interested in medical research %(n/n_valid_) |
| Direct and reliable contact person at the university * | 94.4 (167/177) | 84.7 (100/118) |
| Low effort (timewise) for the practice team | 92.7 (166/179) | 91.1 (112/123) |
| Low effort (timewise) for me as a medical doctor | 91.1 (163/179) | 93.5 (116/124) |
| Compact updates on practice-relevant topics | 89.8 (159/177) | 82.6 (96/116) |
| Timely and practical processing of study results for participating practices ** | 83.1 (148/178) | 69.0 (80/116) |
| Seasonal adjustment to the practice workload ** | 81.8 (144/176) | 65.5 (78/119) |
| Training opportunities in research * | 63.1 (113/179) | 50.4 (60/119) |
| Access to the shared, anonymized project database ** | 60.8 (107/176) | 37.6 (44/117) |

| **Supplement Table 4** Potential barriers regarding an involvement in medical research – comparison between physicians with and without interest in participating in medical research.  (note: percentages of participants who 'rather agree' or 'completely agree' with the presented statements, versus 'rather disagree' and 'completely disagree';  (n_valid_)All = (n_valid_)interested in medical research + (n_valid_)not interested in medical research + missing values;  group differences between interested and not interested in medical research are indicated as follows: *p<0.05, **p<0.005) | | |
| --- | --- | --- |
| Variable | Interested in medical research %(n/n_valid_) | Not interested in medical research %(n/n_valid_) |
| Increase of their daily working time ** | 86.0 (154/179) | 96.2 (127/132) |
| Stressing the doctor’s time too much ** | 71.5 (128/179) | 95.4 (125/131) |
| Putting too much time pressure on the practice team ** | 70.9 (127/179) | 96.2 (126/131) |
| Diminishing the number of patient treatments ** | 48.0 (86/179) | 71.9 (92/128) |
| Disruption of their working routine ** | 46.1 (82/178) | 73.8 (96/130) |
| Fear of insufficient scientific skills | 31.5 (56/178) | 38.6 (49/127) |
| Fear of insufficient current knowledge | 30.2 (54/179) | 34.6 (44/127) |
| Financial losses by participation * | 9.6 (17/177) | 19.0 (24/126) |
