# Supplementary figures and images for: General practitioners’ willingness to participate in research networks in Germany
Source: Scand J Prim Health Care. 2022 Jun 30;40(2):237–45. doi: 10.1080/02813432.2022.2074052 (PMC9397419; doi:10.1080/02813432.2022.2074052)

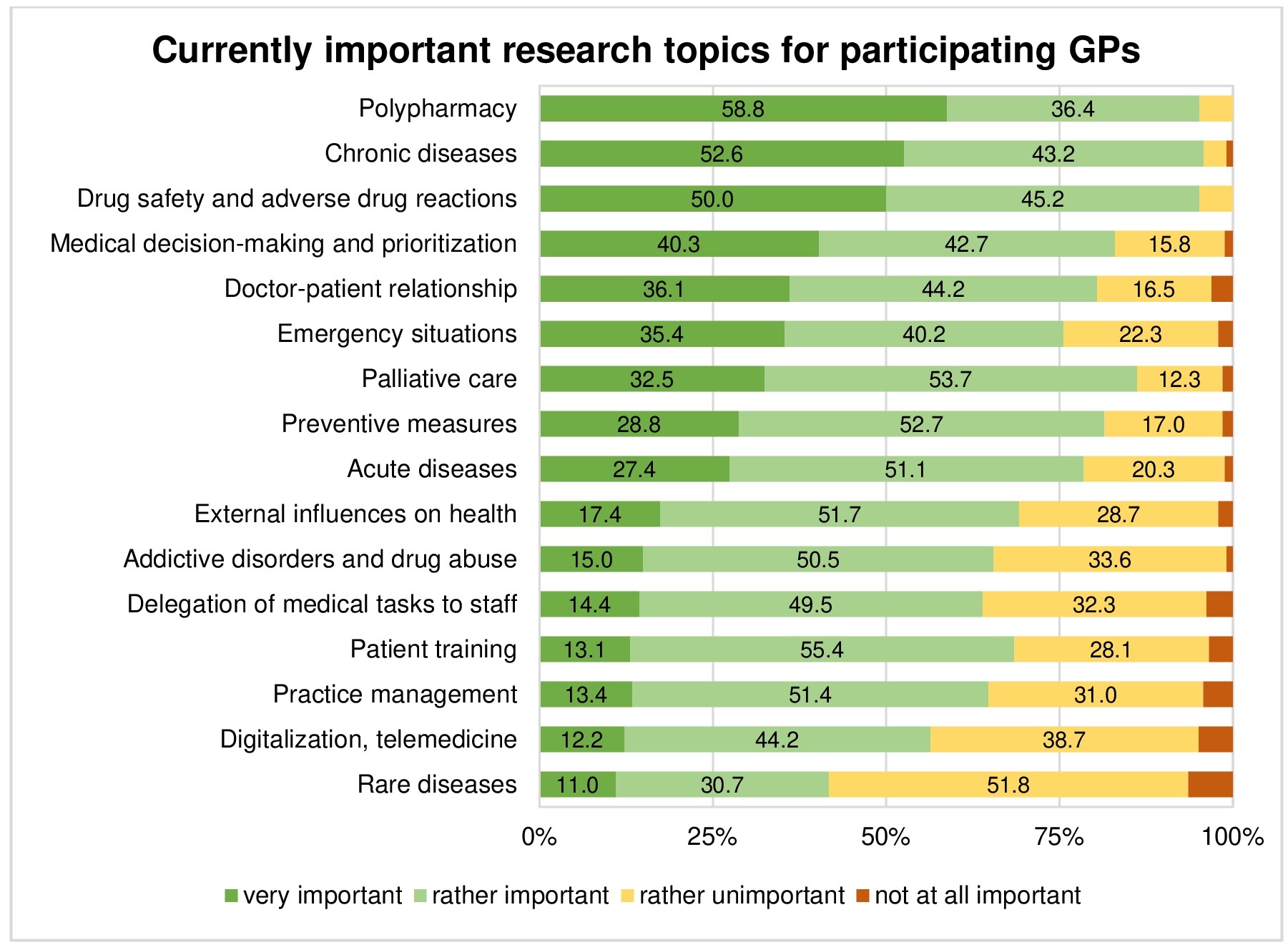

Supplement: Supplemental Material [file IPRI_A_2074052_SM1877.jpg]
